# Compliance With the British Association of Urological Surgeons (BAUS) Standards on Ureteric Stent Documentation: A Retrospective Clinical Audit

**DOI:** 10.7759/cureus.99169

**Published:** 2025-12-13

**Authors:** Amgad Elmadani

**Affiliations:** 1 Urology, Kettering General Hospital NHS Trust, Kettering, GBR

**Keywords:** audit, database, electronic, forgotten ureteric stents, governance, patient safety, quality improvement, ureteric stent, urinary tract stones, urology

## Abstract

Ureteric stents are routinely used in urological practice, but incomplete documentation can result in delayed removal and avoidable complications. This retrospective clinical audit evaluated compliance with BAUS standards before and after the introduction of a new electronic stent-tracking database at a UK district general hospital. All ureteric stents inserted between 20 January and 20 May 2024 were identified from theatre records, with documentation cross-checked against the handwritten stent logbook (pre-implementation) and both the logbook and electronic database (post-implementation). A total of 123 stents were inserted: 55 pre-implementation and 68 post-implementation. Pre-implementation completeness in the logbook was 98.2% (54/55). Post-implementation completeness was 97.1% in both the electronic database (66/68) and the logbook (66/68), with neither system achieving full capture. Missed entries were associated with workflow variation, inconsistent use of parallel systems, and failure to capture antegrade stents. The introduction of the electronic database did not improve overall documentation completeness compared with the traditional logbook, at least at its initial phase. Future improvements should focus on eliminating dual-pathway documentation, ensuring mandatory database use, optimising system configuration for all stent types, enhancing staff training, and re-auditing following implementation of these changes to achieve the target of 100% documentation.

## Introduction

Ureteric stents are essential devices in modern urological practice and are widely used to relieve ureteral obstruction caused by stones, strictures, malignancy, or postoperative complications [1]. Their insertion is one of the most common urological interventions performed globally, with a continuous rise driven by increasing rates of urolithiasis and endourological procedures [2]. Although ureteric stents are effective in restoring drainage and preventing renal deterioration, their use is associated with significant risks if not removed or exchanged within recommended time frames. Forgotten ureteral stents (FUS) may lead to encrustation, infection, fragmentation, obstruction, and even loss of renal function, contributing to preventable morbidity and substantial healthcare burden [3].

Ensuring accurate documentation and reliable tracking of stents is, therefore, crucial to minimise avoidable complications. National bodies have highlighted deficiencies in stent surveillance systems. The Healthcare Safety Investigation Branch (HSIB) reported that delayed stent removal often results from inconsistent documentation and fragmented tracking pathways rather than device-related issues [4]. The British Association of Urological Surgeons (BAUS) similarly emphasised the need for improved governance and robust monitoring as part of wider efforts to enhance patient safety [5].

Evidence from the literature supports the development and adoption of digital solutions to prevent oversight. Systematic reviews demonstrate that forgotten stents are frequently linked to gaps in follow-up processes and poor information continuity [6]. Studies evaluating digital registries have shown reductions in overdue stent removals by improving visibility and automated reminders [7], and smartphone-based applications have been shown to enhance compliance and reduce FUS incidence by promoting better communication between clinicians and patients [8]. Other centres have implemented barcode-based or computerised tracking tools with favourable outcomes, including faster identification of overdue stents and increased documentation accuracy [9].

Despite increasing adoption of digital systems, the transition away from paper-based logbooks remains challenging in many hospitals due to varied staff engagement, parallel workflow use, and inconsistent data entry practices. Quality improvement literature has repeatedly shown that human factors, training gaps, and workflow misalignment are major barriers to digital system effectiveness [10-12]. The National Institute for Health and Care Excellence (NICE) recommends structured audit cycles and system refinement when implementing new digital governance processes to ensure sustained improvement [13].

Given these national reports and published evidence, local audit remains vital to ensure that newly implemented tools achieve their intended safety objectives. Prior studies examining ureteric stent documentation have reported substantial variability in performance, with some centres achieving high levels of capture while others highlight persisting missed entries and lack of integration with coding or clinical systems [14-16]. The need for continuous evaluation is further reinforced by international data indicating that even well-designed systems may fail to achieve complete documentation without strong multidisciplinary engagement and periodic re-auditing [17,18].

This clinical audit, therefore, aimed to evaluate the completeness of ureteric stent documentation before and after the introduction of a new electronic stent database at a single UK district general hospital. By comparing the performance of the traditional handwritten stent logbook with the newly implemented digital system, we sought to assess whether the electronic pathway improved documentation accuracy and to identify ongoing gaps requiring further action.

## Materials and methods

Study design and setting

This was a retrospective clinical audit conducted at Kettering General Hospital, a district general hospital in the United Kingdom. The audit evaluated compliance with local expectations and BAUS recommendations that all ureteric stents should be reliably documented and tracked until removal. The project was registered with the hospital governance department (project code Uro/SE/2024-25/03) as a service evaluation and clinical audit. No patient-level interventions were undertaken, and no identifiable data were retained in the audit dataset, so formal research ethics committee approval was not required, in line with national clinical audit guidance.

Case identification and data sources

The audit included all patients who underwent ureteric stent insertion between 20 January 2024 and 20 May 2024. Cases were identified from theatre management systems and operation notes using procedure codes and text search terms relating to ureteric stent insertion, exchange or change. These theatre records, together with the operative notes, were treated as the reference standard for confirming that a stent procedure had actually been performed.

For each identified case, documentation was checked in two separate systems: (i) the existing handwritten stent logbook, maintained in the relevant theatres; and (ii) the new electronic stent database, which became the standard documentation pathway on 20 March 2024.

The pre-implementation period (paper logbook only) was defined as 20 January 2024 to 19 March 2024. The post-implementation period (parallel paper and electronic systems) was defined as 20 March 2024 to 20 May 2024. For each stent procedure, the audit recorded whether there was a corresponding entry in the handwritten book, the electronic database, both systems, or neither. No demographic, diagnostic, or outcome data were collected beyond brief free-text descriptors of the procedure, speciality, and stent route (retrograde versus antegrade).

Outcome measures

The primary outcome was completeness of stent documentation, expressed as the proportion of stent procedures recorded in each system relative to the total number of stents inserted in the relevant time period. Three primary proportions were calculated: (i) pre-implementation completeness in the handwritten stent book; (ii) post-implementation completeness in the handwritten stent book; and (iii) post-implementation completeness in the electronic stent database.

Secondary descriptive outcomes included the number and proportion of missed entries in each system and qualitative identification of patterns in missed documentation (for example, antegrade versus retrograde approach, or urology versus non-urology specialities). These patterns were derived from free-text descriptions of the procedures in the theatre records and the operative notes.

Data analysis

Data were entered into a simple spreadsheet and analysed descriptively. Categorical variables are presented as counts and percentages, rounded to one decimal place where appropriate. For key proportions such as documentation completeness, numerators and denominators are presented explicitly to facilitate transparent interpretation in keeping with Healthcare Quality Improvement Partnership (HQIP) recommendations for audit reporting. Given the relatively small number of stent insertions and the single-centre design, no formal hypothesis testing or multivariable analysis was undertaken. The intention of this audit was to measure performance against an implicit standard of 100% documentation rather than to test a specific statistical hypothesis.

## Results

Overall stent activity

Over the four-month audit period, a total of 123 ureteric stent insertions were identified from theatre records and operation notes. Of these, 55 procedures occurred in the pre-implementation period, when documentation relied solely on the handwritten stent logbook, and 68 procedures occurred in the post-implementation period, when both the logbook and the electronic stent database were in use (Table 1).

**Table 1:** Summary of stent insertions and documentation completeness

	Number of Stent Insertions	Documentation System	Number Documented	Completeness (%)	Notes
Pre-implementation (20 Jan – 20 Mar 2024)	55	Handwritten stent book	54	98.20%	One missed entry; no electronic system in use
Post-implementation (20 Mar – 20 May 2024)	68	Electronic stent database	66	97.10%	Antegrade stents not captured; some retrograde omissions
Post-implementation (parallel paper log)	68	Handwritten stent book	66	97.10%	Paper system continued concurrently; similar performance to database
Overall (entire audit period)	123	Combined systems	120	97.60%	All procedures correctly coded in theatre records

Pre-implementation completeness (handwritten stent book)

In the pre-implementation period, 55 stent insertions were confirmed in theatre records. Fifty-four of these procedures had a corresponding entry in the handwritten stent logbook, giving a documentation completeness of 98.2% (54/55). One stent insertion (1.8%) was not recorded in the logbook despite being clearly documented in the operation note and correctly coded in the theatre system (Table 1). The missed case occurred in the context of routine urological stone surgery and did not relate to a specific change in personnel or workflow.

Post-implementation completeness (parallel handwritten stent book and electronic database)

In the post-implementation period, 68 stent insertions were identified. The handwritten logbook documented 66 of these 68 procedures, corresponding to a completeness of 97.1%. Two procedures (2.9%) were not recorded in the logbook despite accurate documentation in theatre notes (Table 2).

**Table 2:** Documentation status of stents in the post-implementation period (n = 68)

Documentation Status	Number of Stents (n)	Percentage of Post-implementation Stents (%)
Documented in both logbook and database	64	94.10%
Documented in logbook only	2	2.90%
Documented in database only	0	0%
Not documented in either system	2	2.90%

Over the same period, the electronic stent database also contained entries for 66 of the 68 stent insertions, again yielding a completeness of 97.1% when the database is considered in isolation. Two procedures (2.9%) were not captured electronically. In both of these cases, the stent insertion had been correctly coded and described in the theatre records, confirming that the failure lay in data entry into the tracking system rather than in surgical documentation (Table 2).

Interestingly, the audit concluded that stents missed by the electronic database were generally recorded in the logbook, whereas a very small number of stents were omitted from both systems (Table 2).

Patterns in missed documentation

A qualitative review of the missed entries highlighted two recurring patterns. First, the electronic database did not capture any antegrade ureteric stents during the audit window, even when these procedures were documented in theatre notes and coded correctly. In at least one case, an antegrade stent inserted via nephrostomy was recorded in the handwritten logbook but had no corresponding entry in the electronic registry, indicating a configuration or workflow gap for non-standard access routes (Table 3).

**Table 3:** Patterns in missed documentation by route of insertion and speciality

Stent Type	Typical Documentation Pattern	Identified Issues
Retrograde stents (urology)	High overall documentation across both systems; majority captured in both logbook and database.	Occasional isolated misses due to omission at point of entry, parallel system use, and workflow variability.
Retrograde stents (non-urology)	Generally well-documented in both systems, especially when listed clearly in operation notes.	Variability in familiarity with the stent pathway; inconsistent use of the electronic database.
Antegrade stents	Frequently missing from the electronic database despite being recorded in theatre notes and usually in the paper logbook.	Database not configured to capture antegrade procedures; pathway gaps; absence of prompts for non-urology teams.

Second, in the post-implementation period, a small number of retrograde ureteric stent insertions were documented in theatre records but were not entered into the stent database, despite the system being available and used for other cases on the same operating lists (Table 3). These omissions suggest intermittent lapses in data entry linked to human factors and competing intraoperative or postoperative priorities, rather than a consistent structural flaw in the database itself.

Additional observations from the audit indicated that documentation was generally robust for stents inserted by non-urology specialities, including gynaecology and general surgery, once staff were familiar with the logbook and database (Table 3). Conversely, the absence of systematically configured pathways for antegrade procedures meant that these were more vulnerable to being missed, even when performed by experienced endourological teams.

## Discussion

This audit examined the completeness of ureteric stent documentation before and after implementation of an electronic tracking database and demonstrated that digitisation alone did not improve documentation accuracy. Despite achieving high completeness (>97%) in both periods, neither the handwritten logbook nor the electronic system reached full capture of stent insertions, reaffirming that even well-designed documentation tools may fail without optimised workflows, adequate training, and clearly defined responsibilities. These findings reflect broader evidence that ureteric stent safety is limited less by clinical technique and more by system-level processes, particularly those relating to documentation and follow-up [1-4].

The observed documentation gaps are clinically significant because missed entries pose a recognised risk for forgotten ureteric stents (FUS), which can lead to serious morbidity, including infection, encrustation, obstruction, fragmentation, and loss of renal function [1,3]. The long-term complications of retained stents have been repeatedly highlighted in reviews and case series, including the seminal systematic assessments that demonstrate the correlation between dwell time and stent encrustation [6]. Preventing these outcomes depends primarily on ensuring each stent is documented at the time of insertion, linked to an explicit removal plan, and reliably tracked until removal, a theme that emerges consistently across the literature [2,3,6].

National investigations underscore the importance of robust documentation systems. HSIB’s report on delayed ureteric stent removal demonstrated how inconsistent recording, lack of system integration, and unclear ownership contribute directly to stent retention [4]. The BAUS response further emphasised a national commitment to strengthening governance and the need to move toward structured digital solutions supported by audit, training, and safety-netting measures [5]. Within this context, our findings indicate that the introduction of a digital registry aligns with national recommendations but that without refinement and staff engagement, such systems may not deliver their expected benefits.

The incomplete capture of antegrade stents in the database represents a specific and important gap. The literature on FUS consistently identifies non-standard pathways, such as antegrade insertions, emergency procedures or cases outside core urology, as particularly susceptible to tracking failures [6-8]. The fact that these cases were documented in theatre notes but not transferred into the electronic database suggests a configuration or workflow gap, consistent with reports describing how digital systems can inadvertently exclude atypical or low-volume but clinically important procedure types [7]. Addressing such gaps requires both technical modifications (such as mandatory data fields for antegrade access) and clear training for both urology and interventional radiology teams.

Digital stent registries have shown promise in reducing forgotten stents, but the literature also makes clear that their success is dependent on consistent use. Smartphone-based tracking applications such as those evaluated by Ulker et al. demonstrate improved follow-up adherence and reduced missed removals, but only when staff enter data reliably at the time of the procedure [8]. Similarly, computer-based and algorithm-driven electronic medical record (EMR) systems have been shown to reduce overdue stents by integrating reminders and automated tracking, yet these systems also rely on accurate initial documentation [9]. Internationally, a range of digital innovations, from barcode-based tools to cloud-linked registries and patient-facing apps, demonstrate improved outcomes only when embedded in well-defined workflows [10-12].

Notably, the human-factors literature demonstrates that digital systems fail when they are poorly aligned with real clinical workflows, inconsistently used, or implemented without adequate staff training [11]. A strong body of evidence shows that double-entry systems, voluntary fields, or optional logging decrease compliance, whereas a single, compulsory documentation pathway increases reliability [11,12]. This directly aligns with the finding that maintaining both the handwritten logbook and the electronic database in parallel may have diluted accountability and contributed to non-uniform documentation behaviour. NICE advises that duplication of pathways introduces avoidable variation, increases cognitive load, and should be eliminated during process improvement to enhance reliability [13].

In this audit, staff appeared comfortable with both systems, but the lack of a single mandatory process meant that key actions could be unintentionally omitted. This echoes findings from prior audits showing that the presence of a backup logbook or alternative pathway often reduces compliance with the primary system [14]. Even when computers and smartphone systems are available, studies have demonstrated that clinicians frequently revert to familiar legacy processes unless digital pathways are fully embedded and perceived as easier and more reliable [14].

Furthermore, international studies have highlighted that early-phase rollouts of digital solutions often suffer from temporary drops in performance due to staff unfamiliarity, interrupted workflows, and incremental configuration adjustments [15,16]. The same pattern may have influenced local performance here, where the database was newly implemented, and staff were undergoing a period of adaptation. Evidence from endourology centres that adopted computerised stent registries demonstrates that improvements in documentation often emerge only after iterative refinement cycles, usually through repeated clinical audits [8,15].

In addition, literature examining stent encrustation and retention emphasises the importance of system-wide approaches to prevention, including patient education, automated alerts, and multi-layered safety netting, rather than reliance on clinician memory or manual documentation alone [16-18]. Quality-improvement frameworks, including iterative audit cycles, process mapping, and staff retraining, have been shown to significantly improve compliance and reduce FUS rates over time [14-17]. These frameworks support the need for this audit's recommendations regarding streamlined pathways, improved database configuration, and enhanced training.

Taken together, the findings of this audit are consistent with national and international evidence indicating that digital systems are essential components of modern stent tracking but require systemic, human-factor-aware implementation strategies. To achieve the BAUS-recommended ambition of 100% documentation, the department should eliminate the parallel paper logbook, configure the electronic pathway to include mandatory fields for all stent types (including antegrade), integrate alerts with theatre coding or case closure, and deliver structured staff training. As recommended in quality-improvement literature, these changes should be followed by a re-audit to ensure sustained improvement and full compliance with safety standards [15-18].

This audit has several limitations that should be acknowledged. First, it reflects the experience of a single centre over a relatively short four-month period, which may limit the generalisability of the findings to institutions with different workflows or digital infrastructures. Second, the audit relied on retrospective comparison between multiple documentation sources, and although theatre coding and operation notes were used as the reference standard, there remains potential for classification error if any procedures were miscoded or ambiguously recorded. Third, the electronic database was evaluated only during its early adoption phase, when staff familiarity and workflow integration were still developing; performance may improve as the system becomes more embedded. Fourth, parallel use of both paper and digital systems introduced complexity in determining the definitive cause of missed entries, making it difficult to fully isolate system-related failures from human-factor issues. Finally, the audit did not assess clinical outcomes such as delayed stent removal or complications, which limits the interpretation of the direct patient impact associated with incomplete documentation.

## Conclusions

This retrospective clinical audit found that the introduction of an electronic stent database at a UK district general hospital did not improve the completeness of ureteric stent documentation compared with the existing handwritten logbook. Documentation completeness remained high but suboptimal, at 98.2% before implementation and 97.1% for both the logbook and database after implementation, with persistent gaps particularly affecting antegrade stents and isolated retrograde procedures that were not entered into the registry. These findings support national recommendations from HSIB, BAUS, NICE, and Getting It Right First Time (GIRFT) that reliable stent tracking requires not only digital tools but also clear processes, single-pathway documentation, robust configuration, and sustained staff engagement. Local actions arising from this audit include enhancing staff training, revising the database to ensure capture of all stent types, linking registry prompts to theatre coding, and phasing out the parallel paper logbook once the digital pathway is fully embedded. A re-audit is planned after these changes to assess whether 100% documentation can be achieved and sustained.

## References

[1] Damiano R, Oliva A, Esposito C (2002). Early and late complications of double pigtail ureteral stent. Urol Int.

[2] Scales CD Jr, Smith AC, Hanley JM, Saigal CS (2012). Prevalence of kidney stones in the United States. Eur Urol.

[3] Wang X, Ji Z, Yang P (2024). Forgotten ureteral stents: a systematic review of literature. BMC Urol.

[4] (2025). Health Services Safety Investigations Body: Unplanned delayed removal of ureteric stents. HSIB.

[5] (2025). The British Association of Urological Surgeons: BAUS response to HSIB document. https://www.judiciary.uk/wp-content/uploads/2022/09/2022-0218-Response-from-BAUS-HSIB.pdf.

[6] Thomas R (1993). Indwelling ureteral stents: impact of material and shape on patient comfort. J Endourol.

[7] Tahhan O, Hussain A, Ullah A (2025). 638 improving ureteric stent management: implementing a computer-based stent register to enhance patient safety. BJS.

[8] Ulker V, Atalay HA, Cakmak O (2019). Smartphone-based stent tracking application for prevention of forgotten ureteral double-J stents: a prospective study. Int Braz J Urol.

[9] Sancaktutar AA, Tepeler A, Söylemez H (2012). A solution for medical and legal problems arising from forgotten ureteral stents: initial results from a reminder short message service (SMS). Urol Res.

[10] Singh H, Sittig DF (2020). A sociotechnical framework for safety-related electronic health record research reporting: The SAFER reporting framework. Ann Intern Med.

[11] Carayon P, Wetterneck TB, Rivera-Rodriguez AJ (2014). Human factors systems approach to healthcare quality and patient safety. Appl Ergon.

[12] Molina WR, Pessoa R, Donalisio da Silva R (2017). A new patient safety smartphone application for prevention of "forgotten" ureteral stents: results from a clinical pilot study in 194 patients. Patient Saf Surg.

[13] (2025). National Institute for Health and Care Excellence (NICE): Evidence standards framework for digital health technologies. https://www.nice.org.uk/corporate/ecd7.

[14] Sabharwal S, Macaden AR, Abrol N (2014). A novel computer based stent registry to prevent retained stents: will patient directed automated short message service and letter generator help?. Indian J Urol.

[15] Davis NF, Murray G, O'Connor T (2017). Development and evaluation of a centralised computerised registry for ureteric stents: completing the audit cycle. Ir J Med Sci.

[16] Hameed BM, Shah MJ, Naik N (2021). Are technology-driven mobile phone applications (apps) the new currency for digital stent registries and patient communication: Prospective outcomes using Urostentz app. Adv Urol.

[17] Tomer N, Garden E, Small A, Palese M (2021). Ureteral stent encrustation: epidemiology, pathophysiology, management and current technology. J Urol.

[18] Larkin S, Preston D (2015). Where are the stents? A computerized tracking system to eliminate the forgotten ureteral stent. Urol Pract.

